# Advancing Circularity in Multilayer Film Recycling: Balancing Quality and Sustainability

**DOI:** 10.3390/polym17212868

**Published:** 2025-10-28

**Authors:** Milad Golkaram, Rajesh Mehta, Sami Zakarya, Ilkka Rytöluoto, Lucie Prins, Milena Brouwer-Milovanovic

**Affiliations:** 1 Netherlands Organization for Applied Scientific Research (TNO), Princetonlaan 8, 3584 CB Utrecht, The Netherlands; rajesh.mehta@tno.nl (R.M.); milena.brouwer-milovanovic@tno.nl (M.B.-M.); 2 Leygatech, Research and Development Team, ZA de Chambaud, 43620 Saint Romain Lachalm, France; szakarya@leygatech.fr; 3 VTT Technical Research Centre of Finland, Visiokatu 4, FI-33720 Tampere, Finland; ilkka.rytoluoto@vtt.fi; 4 Netherlands Organization for Applied Scientific Research (TNO), Kesslerpark 1, 2288 GS Rijswijk, The Netherlands; lucie.prins@tno.nl

**Keywords:** life cycle assessment, recycling, plastic

## Abstract

Recycling multilayer films (MLFs) presents significant challenges to achieving circularity. Mechanical recycling, solvolysis (chemical recycling), and dissolution (physical recycling) have been introduced in the past with their strengths and weaknesses. This study uses a series of advanced, pilot-scale processes to improve the quality of recyclates. These include Near Infrared/Digital Watermarking (NIR/DW), super-critical CO_2_ decontamination, dissolution, and innovative mechanical recycling techniques (METEOR and multi-nano layering, MNL). Findings from TRL 5–8 pilots show that recycling different MLF compositions with two routes (dissolution-based and METEOR/MNL-based) can improve the overall quality but this comes with a trade-off. Using 10% recycled content from PET/PE and metalized PP films in 2050 could even increase greenhouse gas (GHG) emissions by 21% and 85%, respectively, compared to landfill incineration. However, PE/PA and PE/EVOH films showed GHG reductions of 0.5% and 4%, respectively. Raising recycled content from 0% to 50% can cut GHG emissions by 36%. These results challenge the current 10% recycled content target, advocating for a more ambitious goal of exceeding 25% by 2050 to enhance sustainability.

## 1. Introduction

The European plastic packaging films market is projected to grow from 7.70 million tonnes in 2024 to 8.77 million tonnes by 2029 [1]. Packaging films are mainly (75–90%) composed of polymers such as (linear-) low-density polyethylene (L-/LDPE), high density polyethylene (HDPE), polypropylene (PP), and polyethylene terephthalate (PET). However, the so-called multilayer films (MLFs) are composed of at least one of these polymers in addition to other non-polymeric materials (5–15%) such as aluminum [2,3,4,5,6]. Ceflex recently reported that 59.4% of flexible packaging produced in the Netherlands corresponds to multimaterial films. This value is estimated to be 41%, 51%, and 38.3%, for Germany, France, and the UK, respectively [7]. This leads to challenges during the recycling stage due to the processing requirements of different polymers (i.e.,: melting point, polarity, immiscibility, viscosity, etc.), lack of defined sorting streams for different compositions, and lack of economical motivation [8,9]. This means that even with the ideal scenario, the net recycling rate (from collection to the market) for post-consumer packaging films will not exceed 72% in developed countries such as the Netherlands [10].

Sorting is usually carried out through several steps: reception of inputs, bag opener, ballistic separator, magnet, manual sorting, and Eddy current, which can vary depending on the material recycling facilities (MRF) [11]. At the end of these steps, different bales are produced that are composed of main products such as PET bottles, PE rigids, PP rigids, and mixed bales [12]. It was proposed to expand the collection portfolio to PE films, other films, and mono-polyolefin-based materials. However, this improvement is not sufficient to meet the recycling target of a 50% recycling rate by 2025, and additional improvements in both recycling technologies and packaging design are required [12].

In terms of recycling technologies, recent developments including new technologies such as chemical (pyrolysis and glycolysis) processes [13,14], metal-catalyzed autoxidation [15,16], and physical (solvent-based) recycling show promising results, but they need further development and up-scaling [17,18,19,20]. Delamination using a micro-perforating technique was proposed for MLFs to recycle different polymer layers separately. This technology can be placed after the separation and sorting of plastic waste and the pre-washing process, and is not affected by the presence of inks and surfactants [21]. Advanced mechanical recycling, which benefits from additional sorting, hot washing, improved extrusion, and deodorization, is also proposed with a recovery rate of around 66%, which is similar to conventional mechanical recycling, although the recyclates were not characterized to prove the quality of the output material [22]. Other technologies addressed the presence of ink and its removal from the MLF [23]. Other researchers successfully applied chemical-based washing techniques that both de-ink and delaminate the multilayer films [24].

Currently, downcycling persists on an industrial scale, while in principle it is possible to recycle PP without the loss of mechanical properties. The recycling capabilities of MLFs composed of PET and PP have been examined. The simulations conducted on recycling indicated that the packaging made from PP could be recycled up to ten times while preserving its mechanical properties and functionality. In contrast, the films made from PET became significantly brittle, rendering them inappropriate for reprocessing into products of comparable economic value. The secondary material derived from the PP films was effectively transformed into new films, showcasing the feasibility of closed-loop recycling [25]. Similar results were obtained for PE/PET-based material, where immiscibility resulted in phase separation causing inhomogeneity-induced losses in ductility and toughness [26]. The significance of either a reduction or an increase in material properties is contingent upon the specific application for which the recycled films are intended. For example, PE/PA films may be utilized as technical components, substituting virgin materials, after incorporating up to 30% ash [8]. Therefore, substitutability should be studied carefully. BASF has reported on the recycling of PE/PA6 and PE/PA6,66 films, comparing the results with virgin LDPE [27]. LDPE has a global warming potential of 1.87 kg CO_2_ eq. compared to that of PA6, which is 5.92 kg CO_2_ eq. [28]. This suggests that the utilization of PA-based MLFs, which ultimately serve as a substitute for LDPE after recycling, is not advantageous throughout their entire lifecycle.

A lack of thorough impact assessment methodology on plastic films waste management systems was identified in the past [2]. For instance, an extensive review of 222 lifecycle assessment (LCA) studies focused on solid waste management has shown that LCA results depend strongly on the specifications, composition, and properties [29]. Additionally, the quality of recycled plastic is seldom taken into account in the corresponding articles [22,30,31,32,33,34,35,36].

The circularity of products was investigated in a few studies. Using the Ellen McArthur Foundation Material Circularity Index (MCI), the circularity of the packaging films was reported [37]. However, the quality of recycled products remains a challenge to be accounted for, and is required to shed light to the actual benefits of recycling. A recent study on the recycling of PP plastics compared mechanical and physical recycling of PP; however, it did not account for the quality of recycled plastic and used a 1:1 factor for the substitution ratio because these values are uncertain and product-specific [38]. For instance, 1 kg of mechanically recycled PP can be treated as functionally equivalent to 0.7–1.0 kg of virgin PP, and therefore, 0.7:1 (or simply 0.7) is the so-called substitution ratio.

The objective of this research is to introduce a new recycling framework including the entire value chain of MLFs. This recycling framework is applied to four use cases (PET/PE, metalized PP, PE/PA, and PE/EVOH), which represent around 77% of the market for MLFs [39]. They are printed films and are based on actual waste collected by municipalities in France.

## 2. Materials and Methods

**Figure 1:**
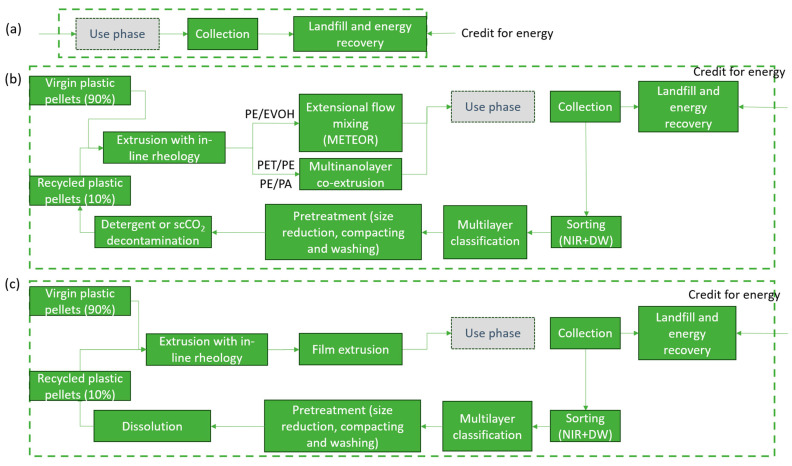
System boundary for the CIMPA Routes and the state-of-the art. (**a**) state of the art, (**b**) CIMPA route using METEOR and (**c**) using dissolution.

The processes involved include collection, enhanced sorting utilizing near-infrared (NIR) technology and digital water marking (DW), decontamination via supercritical CO_2_, extensional flow mixing (METEOR) process for blend homogenization, dissolution for polyolefin (PO) extraction, upgrading through additivation and in-line rheology control, and multi nano-layering (MNL) to enhance barrier and mechanical properties. The NIR/DW technology was chosen, to make sure the correct MLF composition is sorted out to the right technology (Figure 1). Here we refer to two routes known as “CIMPA routes”, including 1 METEOR/MNL (Figure 1b) and dissolution-based (Figure 1c). NIR/DW ensures that metalized films are sorted out to the physical route and the metal fractions are removed through dissolution, while other films are sent to the METEOR/MNL route to improve their properties for their second life in packaging. The details of the sorting process are described in Section S1 in the Supporting Information. Following the sorting, a decontamination technology is required to remove the volatile organic compounds (VOCs). This is especially important when food residues are present in the MLFs at their end-of-life (EoL). To ensure uniform rheological properties, given the variety of polymer grades in the waste, additivation and in-line rheology control is applied. Finally, MNL or METEOR processes are utilized to increase the mechanical properties of the films. The details of the individual processes and the experiments carried out in each step are described in Section S3 in Supporting Information. The recycling processes are evaluated using an innovative life cycle assessment (LCA) method that considers the quality of the recycled film.

Figure 1 illustrates the system boundary for European geography. The use phase is out of scope. Further details on processing steps for each scenario can be found in the Supporting Information Section S4. Based on Global Protocol on Packaging Sustainability 2.0, a typical packaging functional unit for a brand owner or a retailer would be to fulfil packaging functions for 100 g of product from factory to consumer. For a converter supplying packaging film to a customer, the functional unit could be surface area in square meters of a film with a specified performance delivered [40]. The functional unit is considered 1 m^2^ of the films to ensure the comparison of recycled and virgin films takes their functionality into account. The thickness and quality of recycled/virgin blends play an important role in the functionality, and thickness alone does not provide the same unit for comparison. Thus, the FU is the surface area with equal functionality across alternative use cases as recommended by Franklin and associates [41].

The alternative waste treatment technology depends on the composition of the films and whether or not it is technically possible to be adopted. The technologies are in pilot scale and developed by industrial and research partners as stated in the Supporting Information Section S4. The materials were characterized using tensile, dart impact, optical, barrier odor, and migration tests. The detail can be found in the Supporting Information Section S5.

For production of recycled materials, 10% recycled content was used both in CIMPA route and conventional mechanical recycling. Here, conventional mechanical recycling refers to debaling, grinding, washing, drying, extruding, and pelletizing. Material may be washed before grinding, after grinding, or both [41]. The recycled content study was carried out using a co-extruder with various amounts of virgin material. The design description can be found in the Supporting Information (Table S2).

### 2.1. Life Cycle Assessment

There are no sorting fractions for laminates smaller than A4, and they are processed in a mixed-plastic fraction mainly through incineration and landfill [20,42,43]. The amount of packaging in landfills will reduce in the future with more infrastructure for incineration with energy recovery [11]. In the alternative scenarios using advanced sorting and separation technology (NIR and DW), it is assumed that the separately collected films are sent to sorting facility for further recycling (Table S1) while the rest of the films in mixed stream are sent to incineration and landfill. This is estimated to be 35 and 69% for 2017 and 2030 [11] (see Supporting Information Table S1). In this study we assume the statistics from 2017 and 2030 will apply for status quo and future target in 2050, as described by Antonopoulos et al. [11]. Future studies on the MLF EoL forecast can improve the study within modeling inaccuracies. Currently, most studies are performed based on type of polymers and consider MLFs as a bigger group, namely, films. This requires further studies to estimate the material flow of MLFs (<A4) in EU exclusively. Laminates smaller than A4 are studied in the Netherlands but a more comprehensive study for European geography is still missing [42]. For the future scenarios (2050), it is assumed that the environmental profile of virgin plastic production is not changed. All other energy consumptions are based on future (2050) electricity grid.

Ecoinvent database was used as the background dataset unless otherwise stated. For the production of polymers, Industry 2.0 database was used. For the EoL statistics, literature was consulted [11] (Table S1). For pre-treatment, literature was used and adapted for European geography [41]. The details of the life cycle inventory can be found in the Supporting Information (Tables S3 and S4).

### 2.2. Substitution Ratio for the Recycled Plastic

For the quality assessment of recycled plastic and the substitution ratio in the LCA, work done in the past [23,30,31,34,44,45] was used. Huysveld et al. define overall substitutability (OS) as functional of technical substitutability (TS) and market substitutability (MS):

(1) $$OS=\sum_{i=1}^{n}{TS}_{i}\times {MS}_{i}$$

Huysveld et al. define MS as “the potential share of the total market size of the reference virgin material that can be targeted by the recycled material”. The market share is utilized, represented as the weight percentage of each application within the specific plastic market, such as LDPE. The quality of the recycled plastic determines its ability to replace virgin plastic in various applications, depending on their respective market share. The underlying principle is that the quality of recycled plastic is relative to the requirements of each application. For instance, an agricultural film may have stringent technical requirements (e.g., impact strength) but a low market share, whereas a laminate may have less demanding technical needs (e.g., impact strength). To ensure a fair substitutability factor, Equation (1) is employed, considering that the recyclate might be suitable for applications with lower technical requirements but higher market demand. This approach is applied in LCA, following methodologies from previous study by Lisiecki et al.:

(2) $$Q=\sum_{i=1}^{n}w_{i}.f\left({property}^{vir},{property}^{rec}\right),with\sum_{i=1}^{n}w_{i}=1$$

Market share (MS) is the value with which the materials with a specific quality level, Q, have potential to be applied (and thereby substitute virgin material) [46]. W is the weighing factor for each property.

(3) $$Substitutability=\sum_{i=1}^{n}\frac{MS(Q^{rec})}{MS(Q^{Vir})}$$

(4) $$Net\;environmental\;impact=B-\left(S_{vm,1:1}\times TS\times MS\right)$$

Variables are as follows: B, the burdens of sorting, pre-treatment, recycling, and post-treatment; S_vm,1:1_, the savings from avoiding virgin material based on a 1:1 substitution; TS, the technical substitutability of the recycled material [0,1]; and MS, the market substitutability of the recycled material [0,1].

The choice of material properties and their respective importance (weighing factor) is based on interviews with the industrial partners in the project to reduce the bias in the study. The interviews were carried out in semi-structured way where they were asked to provide the most important properties and their importance in RecyQMeter [47].

The impact assessment phase of a life cycle assessment (LCA) focuses on determining the significance of potential environmental impacts based on life cycle inventory (LCI) results. This process typically entails linking inventory data to designated environmental impact categories and their respective indicators, thereby facilitating a better understanding of these impacts.

Life cycle assessment (LCA), as defined by ISO 14040 [48], is a technique used to quantify the environmental aspects and potential impacts associated with a product. LCA consists of four main steps:

1. Goal and scope definition: Establishing the objectives and boundaries of the assessment.
2. Life cycle inventory (LCI): Collecting data and performing calculations to quantify the inputs and outputs of the system under study.
3. Life cycle impact assessment (LCIA): Relating LCI results to environmental impact indicators and categories.
4. Interpretation: Checking for completeness, consistency, sensitivity, accuracy, and uncertainty of the results obtained.

ReCiPe 2016, a comprehensive and harmonized multi-issue LCIA method, which succeeds the Eco-indicator 99 and CML-IA methods, was utilized. ReCiPe 2016 uses eighteen midpoint impact categories (Table S10) to express results at midpoint level.

### 2.3. Limitations

The CIMPA use cases were designed and built using data generated on a lab scale or a small pilot scale, and expert judgement of the CIMPA partners such as film thickness. Any change in the design data, lab testing results, and assumptions of the CIMPA films and selection of the incumbent films by the CIMPA experts will have a direct impact on the LCA results.

Further, testing of film properties was based on samples from lab-sized equipment. Commercial equipment and commercial testing facilities allow better optimization opportunities for design of the films, especially thickness. Therefore, there is an opportunity to optimize the CIMPA film designs at a commercial scale and thereby improve their environmental impacts (LCA).

The environmental impacts during the use phase of the films is excluded as its difficult to assess and is a limitation of the LCA study, considering any change in use phase can have a material influence on the cradle-to-grave environmental impacts of the films.

The LCA study assumes that, at the commercial scale, the CIMPA recycling technologies will perform at same performance level as observed in the CIMPA project. This is a big limitation as sometimes the technologies can perform better or worse during commercialization.

### 2.4. Material Circularity Indicator (MCI)

The assessment of circularity is conducted using Ellen McArthur foundation material circularity indicator. This index was later updated for multilayer material [37]. In this study, the same quality factor will be used in both LCA and MCI estimations. The details of the calculations can be found in the Supporting Information (Section S6 and Tab MCI).

## 3. Results

Recycling enhances the sustainability of plastics [49]. The concept of recycling may appear straightforward; however, the implications of recycling from cradle-to-grave necessitate a thorough examination of the efficiency, energy usage, and quality of recyclates. To explore this issue, pilot-scale studies were conducted to develop more efficient recycling pathways, alongside future electricity grid mixes, ensuring that the carbon intensity of the grid is duly considered [50]. Additionally, quality assessment encompasses not only technical parameters such as tensile strength, dart impact, and tear resistance, but also non-mechanical factors, including odor, transparency, and the migration or the creation of non-intentionally added substances (NIASs). The evaluation of quality adheres to established models that have been widely recognized in previous research [23,30,31,44,51,52]. To ensure that the findings are relevant to the majority of the market for multilayer films (MLFs), four specific use cases have been selected. These include PET/PE, PE/EVOH, PE/PA, and metalized film (metalized PP), which were collected from post-consumer (PET/PE, PE/PA, and metalized film) and post-industrial waste (PE/EVOH) in France.

### 3.1. Quality of Recycled Plastic

The films categorized as post-consumer (PE/PET, PE/PA, and metalized BOPP) and post-industrial (PE/EVOH) were collected and sorted utilizing DW-NIR technology. Prior to the sorting process, a portion of the films was marked with DW technology, as the films currently available in the market do not possess this signature. The sorting results indicated an efficiency rate ranging from 71% to 95%. Subsequently, a pre-treatment process was conducted, which involved washing, shredding, and drying the films.

The PE/PA and PE/PET films, after undergoing pre-treatment, were directed to the decontamination phase, during which supercritical CO_2_ was employed to eliminate volatile organic compounds (VOCs). Subsequently, the flakes were upgraded through compounding with in-line rheology control, followed by the multi-nano layering (MNL) process. This MNL process is implemented to enhance the mechanical and barrier characteristics of the films. The resulting products were utilized as a replacement for virgin PO in the market for films. The quality score is estimated based on the properties of the films (Table S9) and Equation (1). This results in a value of 0.38 for PE/PET and 0.86 for PE/PA. According to the definition provided by [23], 0.38 is “unsuitable” and 0.86 is “good” quality. This means that, with minor improvements, PE/PA can be used as secondary plastic to replace virgin PE/PA, while PE/PET recyclates do not pass the requirements for recyclability. The films performed well with respect to mechanical properties (tear, dart impact, and tensile), and the migration tests showed little traces of intentionally and non-intentionally added substances (IASs and NIASs) diffusing to food. However, despite the acceptable gloss and water vapor transmission rate (WVTR), the haze and oxygen transmission rate (OTR) did not show high performance (Table S3). This led to a low quality value, especially for PE/PET.

The pre-treatment phase for metalized films occurred subsequent to a dissolution-based recycling process, during which the polyolefins (POs) were selectively recovered from the complex multilayer waste and thereafter re-stabilized and upgraded through compounding with in-line rheology control. These materials are suitable for film extrusion as substitutes for PO films. Recently, it was reported that the use of dissolution–precipitation (or solvent-based recycling) can produce high-quality recyclate and can reduce GHG emissions [38]. The study did not take the quality into account and assumed that dissolution must have a high substitutability potential. In fact, the study used a substitution potential of 1:1. For CIMPA demonstrators, the mechanical properties of the films were superior to other films; however, due to the presence of traces of metals and other contaminants, the haze was too high to be used for transparent films, leading to an overall substitutability of 0.4. This means that an additional filtration step is required.

Finally, the PE/EVOH films followed the same path as PE/PET and PE/PA films, except that instead of using MNL, they were sent to the extensional flow mixing process (METEOR). The METEOR process is a process where the polymer blends are oriented under control and micro phases are formed, potentially improving the barrier and mechanical properties. The result of the characterization showed a similar trend as the other films, with all but haze and OTR contributing to an increase in quality score (0.4). s in haze after recycling have been reported in the past [53]. The aesthetic appeal of packaged goods can be affected by this element, especially when transparency is crucial for drawing in customers. Recycled EVOH films may be more appropriate for uses where visibility requirements are not as strict, such as packaging that includes labels or is designed for opaque applications [53].

### 3.2. Assessment of Recycling Routes and Their Environmental Impact

This chapter presents the findings for the product system under investigation. The results are provided for the previously established functional unit (FU) of 1 square meter of packaging film. To evaluate the overall environmental performance of the packaging films, the ReCiPe midpoint (H) method is employed.

**Figure 2:**
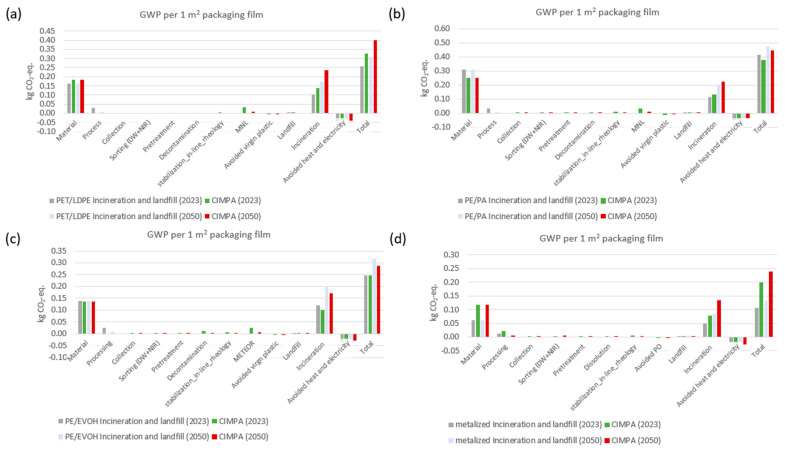
Result of impact for 1 m^2^ film using state-of-the-art waste treatment technology compared with CIMPA route. ReCiPe 2016 midpoint (H) method was used. The results shown different MLF compositions: (**a**) PET/LDPE, (**b**) metalized, (**c**) PE/EVOH and (**d**) PE/PA.

**Figure 3:**
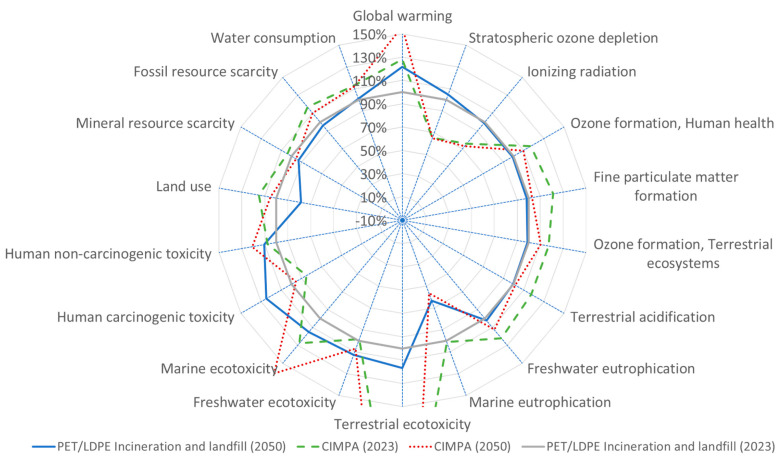
Spider plot representation of impact assessment result for 1 m^2^ film (PET/PE) using state-of-the-art waste treatment technology compared with CIMPA route. ReCiPe 2016 midpoint (H) method was used.

Figure 2 illustrates the outcomes associated with each process in the lifecycle of PET/PE films. The thickness of the incumbent film is less than that of CIMPA products (82 µm compared to 97.5 µm), while the weight percentage of PET in CIMPA products is lower (21% versus 10%). The interplay between composition and thickness is reflected in the material emissions depicted in Figure 2. The material emissions for CIMPA are 13% greater than those of the incumbent for every square meter of film produced. The total emissions for PET/LDPE films, considering only incineration and landfill as end-of-life treatment methods, are estimated at 0.26 kg CO_2_ equivalent. This figure is projected to rise by 0.31 by the year 2050 due to a decrease in the landfill fraction (as shown in Table S1). When utilizing the CIMPA solution, greenhouse gas emissions are estimated at 0.33 kg CO_2_ equivalent. If the anticipated electricity grid mix for 2050 is applied, along with a reduced landfill/incineration ratio (refer to Table S1), this value increases to 0.40 kg CO_2_ equivalent. In terms of other impact categories (illustrated in Figure 3), the findings indicate that, with few exceptions, the CIMPA approach incorporating 10% recycled content does not outperform the incineration and landfill scenarios. This is primarily attributed to the decline in quality associated with the use of 10% recycled content. Additionally, the thickness of films produced from recycled materials is considerably greater than that of incumbent films, resulting in increased material demand and incineration at the end of their lifecycle.

The analysis of GWP for PE/PA films reveals that the CIMPA route demonstrates superior performance compared to the state-of-the-art scenario (landfill and incineration) in both the current year and the year 2050, with improvements of 7% and 4%, respectively, as illustrated in Figure 2. This variance is primarily attributed to differences in material and incineration emissions. Specifically, the composition of the two film types differs, with PA constituting 30% in the state-of-the-art route and only 15% in the CIMPA route. Consequently, the films currently available in the market exhibit a higher GWP than those produced through the CIMPA process.

**Figure 4:**
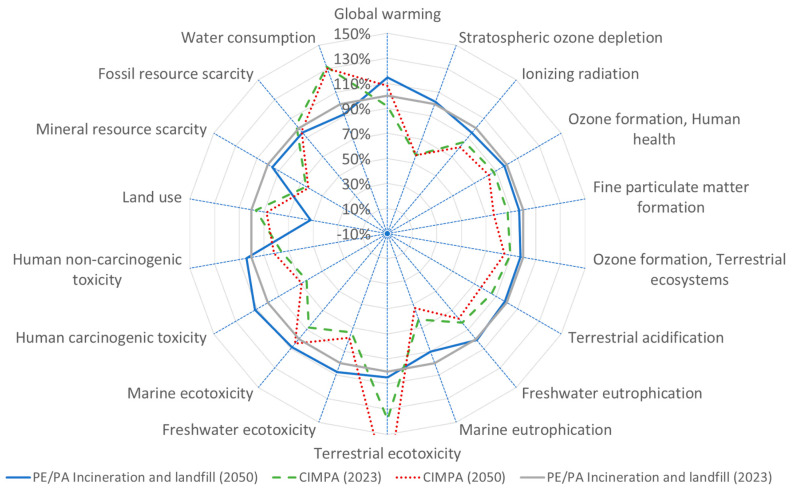
Spider plot representation of impact assessment result for 1 m^2^ film (PE/PA) using state-of-the-art waste treatment technology compared with CIMPA route. ReCiPe 2016 midpoint (H) method was used.

**Figure 5:**
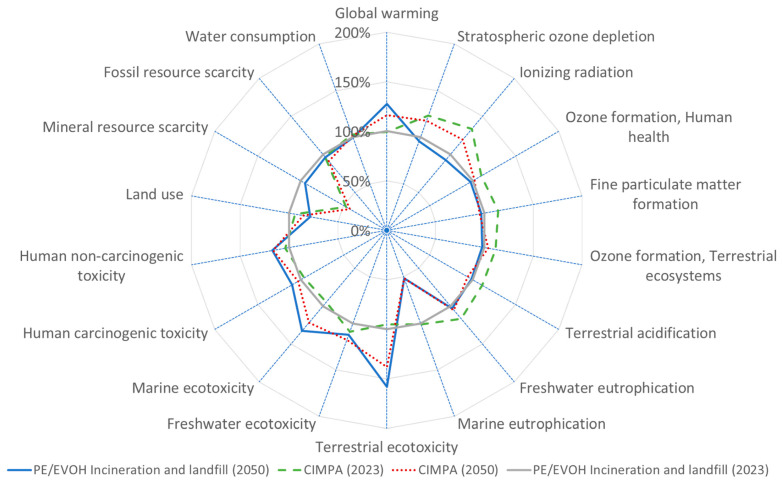
Spider plot representation of impact assessment result for 1 m^2^ film (PE/EVOH) using state-of-the-art waste treatment technology compared with CIMPA route. ReCiPe 2016 midpoint (H) method was used.

**Figure 6:**
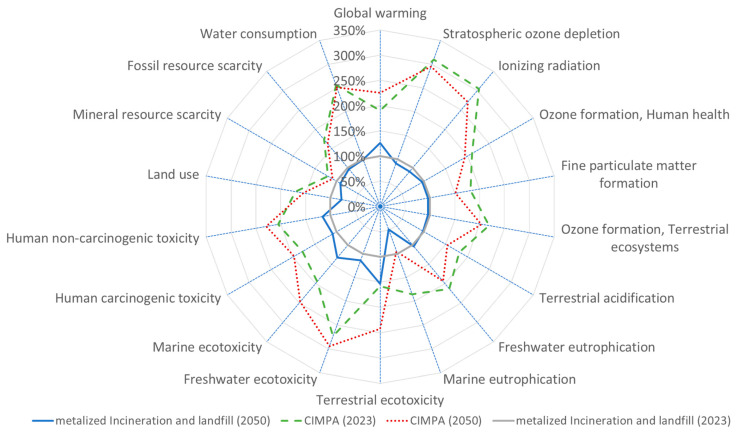
Spider plot representation of impact assessment result for 1 m^2^ film (metalized) using state-of-the-art waste treatment technology compared with CIMPA route. ReCiPe 2016 midpoint (H) method was used.

Figure 3, Figure 4, Figure 5 and Figure 6 present results across 17 other impact categories, where CIMPA either outperforms or is comparable to other methods, with the exception of land use, terrestrial ecotoxicity, and water consumption. The reduction in land use in the 2050 scenarios is due to a 10% decrease in landfill usage, while incineration requires less land than recycling. A similar trend is observed for water consumption and terrestrial ecotoxicity, where recycling is less favorable than both incineration and landfill.

In a manner akin to the results observed for PE/PA films, the findings for PE/EVOH indicate that the CIMPA route surpasses the state-of-the-art (SoA) in terms of GWP by 9% in 2023 and by 0.5% in 2050, as illustrated in Figure 2. Nevertheless, a trade-off is evident in other impact categories, with eight categories demonstrating better performance for the CIMPA route.

For BOPP films recycled through the dissolution method, Figure 2 demonstrates that the CIMPA solution exhibits a GWP that is 82% and 85% greater than that of landfill and incineration methods for the years 2023 and 2050, respectively. This increased GWP is attributed to the greater thickness of the films, measuring 40 µm for the state-of-the-art (SoA) and 70 µm for CIMPA, which results from the film extrusion process following the upgrading with in-line rheology control. This issue could have been mitigated if the films are produced at a full industrial scale with in-line control of thickness during the extrusion. This will be improved since the dissolution process has shown to have the best quality, being close to virgin materials. Initial trials showed that, by additional filtration during scale-up, the haze is improved and white powders are formed, suggesting closed-loop recycling (see Supporting Information Figure S3).

## 4. Discussion

Using 1 m^2^ as the functional unit of the study and accounting for the functionality of the produced materials and their virgin equivalents, thickness plays an important role. In this study, the substituted films were assumed to be 50 µm thick based on the average data from the technical datasheets. The films had approximately 10% recycled content following the plastic packaging waste directive (PPWR) [54]. To assess whether this amount is desirable, a sensitivity analysis is carried out to check the effect of the recycled content on the environmental impact of the recycling route.

**Figure 7:**
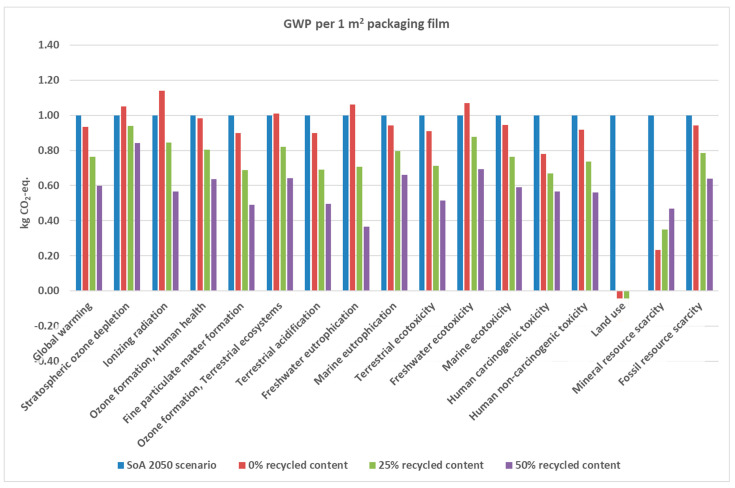
Optimization of recycled content and its impact on the GHG emissions of the films.

The influence of varying levels of recycled content is illustrated in Figure 7. Based on the quality of recycled plastic utilized in PE/EVOH demonstrator with differing recycled contents (0, 25, 50%), a substitution ratio is calculated (see Supporting Information Table S5). It is important to note that the substitution ratio ranges from 0 to 1, reflecting the quality of the final product and its market application share. Figure 4 indicates that an increase in recycled content generally results in a reduced impact across all categories, except for land use and mineral resource scarcity. Furthermore, the data reveals that achieving a recycled content of 25% is sufficient to diminish impacts below those of the SoA across all impact categories.

RecyClass has recently issued guidelines aimed at enhancing the recyclability of multimaterial plastic packaging. While these recommendations have the potential to support the industry’s advancement towards a more circular economy, they require a more thorough evaluation to demonstrate their sustainability. A recent study indicated that, in a specific application, namely nuts packaging made from PET/PE, transitioning from an MLF to an all-PE alternative resulted in a 31% increase in GHG emissions [55]. This originates from the thickness of the materials used in this study.

The incorporation of recycled materials follows a similar trajectory; integrating recycled content is not merely a matter of increasing the amount of recycled plastic in the conversion. It necessitates a quality level that is sufficiently high (i.e., greater than 0.9) to effectively substitute virgin materials. The selection of the virgin plastic that the recycled film is intended to replace significantly influences the environmental impact of the packaging throughout its lifecycle. A balance is often achieved by considering the percentage of virgin material in the virgin/recyclate mixture, the energy consumed during reprocessing, the material losses incurred during this process, and, importantly, the quality of the final product. Therefore, what is the optimal level of recycled content?

The response depends on the nature of the question posed. Regarding material circularity, it was determined that incorporating a greater amount of recycled content results in an increased circularity index. For instance, in the case of the PET/PE demonstrator examined in this study, the circularity indicator for the initial lifecycle is determined using the Material Circularity Indicator (MCI) established by the Ellen MacArthur Foundation. The MCI is estimated to be 0.29, which aligns with previously reported values found in the literature [37]. For the second life using 10% recycled content, the MCI was 0.33 compared to 0.59 from the literature. The lower value is due to the high recycled content (around 42%) of the reference [37] (details on the calculations are in the Supporting Information Section S6). For the incineration and landfill scenario, the MCI is calculated to be 0. This means that despite the increase in circularity compared to incineration, MLPs require higher recycling rates and recycled content in their second life time.

Plastics Europe calls on policymakers to uphold the 2030 mandatory recycled content targets outlined in the PPWR proposal, which includes a 10% target for contact-sensitive applications. These targets are crucial for facilitating the transition of the plastics industry towards circularity and climate neutrality [54]. Through the comprehensive analysis conducted in this study, we demonstrate that, despite an increase in circularity, a mere 10% recycled content in packaging plastics does not inherently equate to environmental sustainability. In fact, we advocate for recycled content levels exceeding 25% for MLFs. Further investigation is required to assess this threshold for various applications on an individual basis. Moreover, for non-contact sensitive packaging, PPWR recommends 35% recycled content by 2030. High-quality recyclate that is not food-grade could potentially be used for such applications and thus should not be ignored.

**Figure 8:**
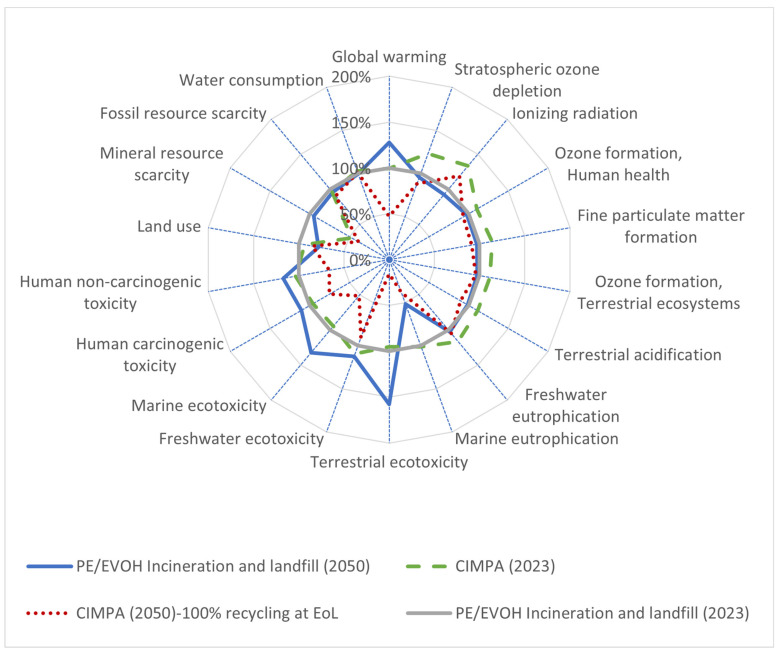
Spider plot representation of impact assessment result for 1 m^2^ film (PE/EVOH) using state-of-the-art waste treatment technology compared with CIMPA route. ReCiPe 2016 midpoint (H) method was used. For 2050 scenario, it is assumed that all of the films at EoL are recycled and no incineration or landfill occurs.

Furthermore, due to PPWR guidelines, it is expected that the recycling rates will increase compared to those of Antonopoulos et al. The exact increase is difficult to predict. Here, we analyze the most optimistic case where all the plastic films are fully recycled and 10% recycled content is incorporated in the new films. Figure 8 demonstrates the results. The global warming potential reduces from 116% (Figure 5—references the SoA scenario) to 47%. This is mainly due to the emission prevention from the incineration of MLFs, while some impact categories unaffected by incineration are not changed significantly (e.g., water consumption and fresh water eutrophication).

This work demonstrates how combining targeted sorting (NIR/DW), supercritical CO_2_ decontamination, METEOR homogenization, dissolution-based separation, and MNL structuring can systematically address the challenges related to MLF recycling. In particular, METEOR reduces phase separation in PE/EVOH by generating controlled microphases that sustain tensile and dart impact performance even at elevated recyclate shares, whereas MNL partially compensates for barrier penalties in blends (e.g., PE/PA) by architecting many thin layers that interrupt defect propagation. Dissolution acts as a selective “quality resetter” for metalized films, recovering PO streams with rheology that is comparable to virgin feeds; our results indicate that haze, rather than mechanical properties, is the inhibiting factor, pointing to inline filtration/polishing as the priority for improvements during scale-up.

Relative to existing data, our PE/PA outcomes align with reports that recycled PA-rich laminates can be used in PO-type applications. For PET/PE and metalized PP, our results add nuance to prior conclusions on “high-quality” solvent routes by showing that optical specifications (haze) dominate application access and hence market substitutability; mechanically “good” recyclate can still underperform environmentally if it forces thicker films or excludes high-volume applications. These comparisons make clear which gaps are addressed here: (i) a validated, quality-coupled substitution framework for films, (ii) pilot-scale evidence linking unit operation choices to functional film properties, and (iii) system-level LCA that reflects both quality and thickness effects.

Broader circularity implications follow directly: raising recycled content above 25% meaningfully improves both MCI and GWP for PE/EVOH and PE/PA archetypes, provided thickness is controlled and quality guidelines are met; for PET/PE and metalized PP, dissolution and improved fines removal (filtration/degassing) is the shortest path to unlock higher-value, transparent applications and thus a higher MS.

## 5. Conclusions

A life cycle assessment (LCA) was conducted to evaluate the environmental impact of CIMPA recycling technologies across 18 impact categories. The study analyzed the entire cradle-to-grave lifecycle, from waste collection to the production of recycled films containing 10% recycled content. Four scenarios were examined for each film demonstrator: two state-of-the-art (current and 2050 projections) and two CIMPA-based scenarios. State-of-the-art scenarios used landfill and incineration, with 53% incineration and 47% landfill in the current scenario, reducing landfill amounts to 10% by 2050. CIMPA scenarios increased recycling rates from 35% to 69% between the incumbent year and 2050, incorporating the 2050 electricity grid mix.

The demonstrators studied included PET/PE, PE/EVOH, PE/PA, and metalized BOPP films. Results showed that, for PET/PE, GHG emissions were 21% higher with CIMPA than with state-of-the-art technologies, increasing to 23% in 2050. Most impact categories showed poorer performance under CIMPA, except for stratospheric ozone depletion, ionizing radiation, and marine eutrophication. Similarly, for metalized PP films, GHG emissions under CIMPA were 82% higher in 2023 and 85% higher in 2050.

Conversely, PE/EVOH films showed a 9% reduction in GHG emissions in 2023 and 0.5% in 2050 under CIMPA, though eight impact categories favored CIMPA. PE/PA films performed better in GWP, with a 7% reduction in 2023 and 4% in 2050. However, CIMPA performed worse in land use, terrestrial ecotoxicity, and water consumption due to the higher resource demands of recycling.

The study highlighted the critical roles of material composition and film thickness. Secondary films performed better if their thickness matched the reference films, and substituting thinner PET or PA layers with LDPE improved functionality. While CIMPA offers potential benefits, trade-offs exist, and its environmental impact depends on material and waste treatment practices.

In summary, the integration of quality-aware substitution into LCA shows that recycled-content targets must be paired with thickness parity and application-specific property compliance to deliver real environmental gains. Practically, our results justify pursuing ≥25% recycled content for non-food MLFs (and 10–20% for contact-sensitive uses subject to migration/barrier limits), alongside scale-up measures that reduce haze and enable access to high-volume applications.

## Data Availability

For the LCA foreground data, the study considers information from specific plant processes as provided in the manuscript. Some of the underlying data we use is protected by an NDA. However, we have used public sources wherever possible to limit the number of datapoints that cannot be shared. As for the background systems, the study relied on datasets from ecoinvent (https://ecoinvent.org/) (accessed on 20 December 2024). RecyQMeter uses a user-friendly interface that is built in RStudio (rstudio 2022.07.1 Build 554) running on the DIAMONDS online platform. Users can request an account via https://diamonds.tno.nl/. Upon approval, the user can then log into the system and use the tool.

## Supplementary Materials

The following supporting information can be downloaded at https://www.mdpi.com/article/10.3390/polym17212868/s1, Figure S1: Sorting steps in alternative scenarios using DW and NIR. The MLFs are separated into different streams and can be sent to recycling through either mechanical or physical route; Figure S2: CIMPA processing scheme on sorted waste sample at lab scale; Figure S3: Process flow diagram of the TNO Möbius dissolution technology as designed for CIMPA; Figure S4: Demonstrators from dissolution experiments. Table S1: Summary of the studied scenarios in LCA films; Table S2: Thickness and composition of the main polymers used in the four demonstrators. Incumbent re-fers to the commercial films and CIMPA refers to the resulting films from CIMPA process; Table S3: Data input for the production of 1m^2^ film. The components include the materials used during the extrusion step; Table S4: Data input for the materials and energy consumption in end-of-life unit processes in the treatment of 1 kg waste film. Data from the project partners during pilot trials; Table S5: Result of substitution ratio for different recycled content amount of PE/EVOH demonstrators; Table S6: MCI results for PET/LDPE sample. The results show the first life cycle; Table S7: MCI results for PET/LDPE sample. The results show the second life cycle. The quality value and recycled input of 90% and 10% are used for CIMPA, respectively. The reference result is based on literature for 42% recycled content as the input to the second life cycle; Table S8: Sources of electricity grid mix for the future scenario (2050) (International Energy Agency (2023)); Table S9: Result of characterization for the four demonstrators; Table S10: The eighteen categories that ReCiPe 2016 used at midpoint level. Refs. [11,18,20,21,37,56,57,58,59,60,61,62,63,64] are cited in Supplementary Materials file.
