# Supplementary material for: Advancing Circularity in Multilayer Film Recycling: Balancing Quality and Sustainability
Source: Polymers (Basel). 2025 Oct 28;17(21):2868. doi: 10.3390/polym17212868 (PMC12610871; doi:10.3390/polym17212868)
Supplement: Supplementary file 1 [file polymers-17-02868-s001.zip › polymers-17-02868-supplementary.pdf]

polymers

*Supporting Information:*

# Advancing Circularity in Multilayer Film Recycling: Balancing Quality and Sustainability

**Milad Golkaram [1,*], Rajesh Mehta [1], Sami Zakarya [2], Ilkka Rytöluoto [3], Lucie Prins [4] and Milena Brouwer-Milovanovic [1]**

[1] Netherlands Organization for Applied Scientific Research (TNO), Princetonlaan 8, 3584 CB Utrecht, The Netherlands; rajesh.mehta@tno.nl (R.M.); milena.brouwer-milovanovic@tno.nl (M.B.-M.)
[2] Leygatech, Research and Development Team, ZA de Chambaud, 43620 Saint Romain Lachalm, France; szakarya@leygatech.fr
[3] VTT Technical Research Centre of Finland, Visiokatu 4, FI-33720 Tampere, Finland; ilkka.rytoluoto@vtt.fi
[4] Netherlands Organization for Applied Scientific Research (TNO), Kesslerpark 1, 2288 GS Rijswijk, The Netherlands; lucie.prins@tno.nl
* Correspondence: milad.golkaram@tno.nl

**Table S1.** Summary of the studied scenarios in LCA films [11].

| Entry | Scenario ID | Year | Electricity grid mix | Incineration | Landfill | Recycling |
|---|---|---|---|---|---|---|
| **1** | SoA 2023 | 2023 | 2023 | 53% | 47% | 0 |
| **2** | SoA 2050 | 2050 | 2050 | 90% | 10% | 0 |
| **3** | CIMPA 2023 | 2023 | 2023 | 35% | 30% | 35% |
| **4** | CIMPA 2050 | 2050 | 2050 | 28% | 3% | 69% |

## Overview of sorting steps

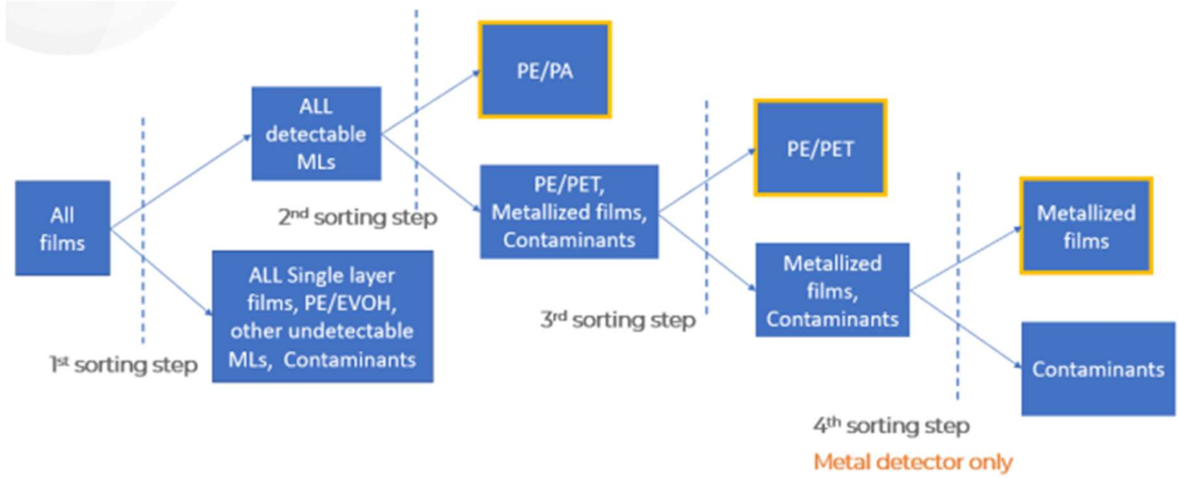


**Figure S1.** Sorting steps in alternative scenarios using DW and NIR. The MLFs are separated into different streams and can be sent to recycling through either mechanical or physical route.

 https://doi.org/10.3390/polym17212868

First sorting step: This first step allows us to concentrate all MLs films into one batch. It means the machine blows upward all PE/PA, PE/PET structures with NIR-based detection and all metallized structures with metal detector at the same time[56]. All mono-materials (PE, PP, PET) and contaminants (everything which is not plastic film) will not be ejected and fall into the negative fraction. All NIR-undetectable structures such as PE/EVOH, PP/EVOH, carbon black containing objects will thus fall into the negative fraction.

Second sorting step: The positive fraction of the first sorting step is taken, and the machine is ordered to blow PE/PA structures only, using NIR-based detection. The contaminants ejected by mistake are mostly other mono-PE structures that can sometimes be mistaken for PE/PA.

Third sorting step: The negative fraction of the second sorting step is taken, and the machine is ordered to blow PE/PET structures only, using NIR-based detection. The contaminants ejected are mostly rigid PE/PET trays that remain from a previous imperfect ballistic sorting. The core of those trays is often rigid PE/PET, and their lids are made of a PE/PET film structure.

Fourth sorting step: The negative fraction of the third sorting step is taken, and the machine is ordered to blow metal-containing objects only using an induction sensor. The contaminants ejected are mostly metallic cans that remain from a previous imperfect ballistic sorting and surgical masks due to their metallic parts. Overview of statistics for scenarios.

## Film compositions

These are the films made from virgin materials used currently in the market.

Demonstrator 1: PET/LDPE (two layers: PET+LDPE)

- The thickness of the PET is 12 microns and LDPE is 70 microns, leading to total thickness of the multilayer film of 82 microns.
- The densities of PET and LDPE are 1400 and 924 kg/m$^3$, respectively.
- The weight of 1 m$^2$ film is therefore 0.08 (0.065+0.0168) kg.

Demonstrator 2: PE/EVOH (four layers: EVOH+LDPE+LLDPE+tie layer):

- The thickness of the EVOH is 4 microns, tie layer is 8 microns, LLDPE is 33.6 microns, and LDPE is 34.4 microns.
- The densities of EVOH, tie layer, LLDPE, and LDPE are chosen as 1190, 918 and 918 and 920 kg/m$^3$, respectively.
- The weight of 1 m$^2$ film is therefore 0.075 kg.

Demonstrator 3: PE/PA (three layers: PE+PA+tie layer):

- The thickness of the PE is 57 microns, PA is 23.8 microns, and the tie layer is 14.3 microns.
- The densities of PE, PA, and the tie layer are chosen as 920, 1140, and 918 kg/m$^3$, respectively.
- The weight of 1 m$^2$ film is therefore, 0.0927 (0.0524+0.0271+0.0131) kg.

Demonstrator 4: PPmetBOPP (two layers: PP+metalizedPP):

- The thickness of the PP is 40 microns and metal is 10 nm.
- The densities of PP and metal are chosen as 918 and 2700 kg/m$^3$, respectively.
- The weight of 1 m$^2$ film is therefore 0.0367 kg.
- The tie layer is made from vinyl acetate.

**Table S1**. Thickness and composition of the main polymers used in the four demonstrators. Incumbent refers to the commercial films and CIMPA refers to the resulting films from CIMPA process.

| | Thickness CIMPA (μm) | Thickness incumbent films (μm) | Composition CIMPA (%) | Composition Incumbent (%) |
|---|---|---|---|---|
| **PE/PET** | 97.5 | 82 | LDPE: 90, PET: 10 | LDPE: 79, PET: 21 |
| **PE/PA** | 94.5 | 95.1 | LDPE: 85, PA: 15 | LDPE: 66, PA:34 |
| **PE/EVOH** | 74.5 | 80 | LDPE: 95 EVOH: 5 | LLDPE:46, LDPE:47 EVOH: 7 |
| **Metalized BOPP** | 70 | 40 | LDPE: 90, PP: 10 | PP:100 |

**Table S2**. Data input for the production of 1m$^2$ film. The components include the materials used during the extrusion step.

| Component | Value | Unit |
|---|---|---|
| | **PET/LDPE** | |
| | Materials | |
| **LDPE** | 0.0647 | kg |
| **PET** | 0.0168 | kg |
| **Tie layer** | 0.002 | kg |
| | Processing | |
| **Film extrusion** | 0.0815 | kg |
| | **PE/PA** | |
| | Materials | |
| **LDPE** | 0.0524 | kg |
| **PA** | 0.0271 | kg |
| **Tie layer** | 0.0131 | kg |
| | Processing | |
| **Film extrusion** | 0.0926 | kg |
| | **PPmetBOPP** | |
| | Materials | |
| **PP** | 0.0368 | kg |
| **Aluminum** | 2.7e-5 | g |
| | Processing | |
| **Film extrusion** | 0.0368 | kg |
| | **PE/EVOH** | |
| | Materials | |
| **LLDPE** | 0.0308 | kg |
| **LDPE** | 0.0316 | kg |
| **Tie layer** | 0.00727 | kg |
| **EVOH** | 0.00476 | kg |
| | Processing | |
| **Film extrusion** | 0.0745 | kg |

**Table S3**. Data input for the materials and energy consumption in end-of-life unit processes in the treatment of 1 kg waste film. Data from the project partners during pilot trials.

| Process | Component | Value | Unit | Comment |
|---|---|---|---|---|
| **NIR** | Electricity | 0.0265 | kWh | |
| **+** | Transportation | 0.000015 | tkm | |
| **DW** | Efficiency | 71-95 | % | |

| | | | | |
|---|---|---|---|---|
| **scCO2** | Efficiency | 91 | % | |
| | Liquid CO2 | 0.050 | kg | |
| | Electricity | 0.4430 | kWh | |
| **VAREX** | LLDPE | 0.0495 | kg | Compatibilizer according to VTT around 1% MAH in the compatibilizer (MAH-LLDPE) |
| | Maleic anhydride | 0.0005 | kg | Compatibilizer according to VTT around 1% MAH in the compatibilizer (MAH-LLDPE) and 10% of compatibilizer in the blend |
| | Pentaerythritol | 0.0040 | kg | |
| | Electricity | 0.300 | kWh | |
| | Efficiency | 100 | % | |
| **METEOR** | Electricity | 0.7 | kWh | |
| | Efficiency | 100 | % | |
| **MNL** | Electricity | 0.7 | kWh | |
| | Efficiency | 100 | % | |
| **Dissolution** | Solvent | 0.0020 | kg | |
| | Heat | 0.4000 | kWh | |
| | Electricity | 0.0020 | kWh | |
| | Efficiency | 90 | % | |
| | non-solvent | 0.0020 | kg | |

Quality calculations are based on quantifiable parameters like tensile strength (ISO 527), tensile modulus (ISO 527), and Charpy impact strength (ISO 179). Processability is determined by the flow behaviour, measured via the Melt Flow Index (MFI) according to ISO 1133. Scoring functions are defined for each relevant property per application or group of comparable applications (e.g., bottle, film). These functions return a value between 0 and 1, depending on how closely the recycled plastic's property matches the typical virgin grades.

The scoring function produces a value close to 1 for recyclates whose MFI (or IV) value falls within the optimal processing range for the specific application. Recycled materials that are challenging to process receive a low score. Materials that cannot be processed with the intended technology receive a score of zero.

Quality is calculated by summing the scores of the essential mechanical properties of the virgin material, weighted by their importance for a specific application. The scoring functions evaluate how undesirable changes in each property are for that application, and both the scoring functions and weights are tailored to the application's requirements. For example, rigid injection moulding tolerates a high modulus, whereas flexible injection moulding does not.

Defining scoring functions is crucial for calculating quality factors, as they assess how well the recycled plastic's properties align with optimal values for specific applications. Several factors must be considered when designing these functions: the optimal value range, whether the property needs to match the virgin plastic exactly or just meet a threshold, and the application's tolerance for deviations. These data can be seen in the Supporting Information Excel documents.

A good scoring function gives a score of 1 when the recycled plastic's property is within the optimal range and a score less than 1 when deviations reduce quality. These functions are shaped by technical data and expert input to reflect the application's sensitivity to deviations, leading to different types of functions:

Trapezoidal: Scores 1 within an optimal range and decreases linearly outside it. The slope's steepness is adjusted based on the importance of the deviation.

Bell-shaped: Ideal for properties that must stay within a strict range, where deviations drastically impact functionality.

Gaussian: Transitions from an optimal range to a single ideal value, useful for converters requiring precise property values.

Single-Sided Trapezoidal and Sigmoidal: Used when only a lower threshold is necessary. Sigmoidal functions provide a more gradual score decline compared to the linear drop in single-sided trapezoidal functions.

For our calculations, a Trapezoidal type is used. These scoring functions are tailored to each property and application to accurately reflect quality loss due to deviations from optimal values.

**Table S5.** Result of substitution ratio for different recycled content amount of PE/EVOH demonstrators.

| Recycled content % | substitutability with FCM | substitutability without FCM |
|---|---|---|
| **0** | 0.99 | 0.99 |
| **25** | 0.59 | 0.99 |
| **50** | 0.57 | 0.96 |
| **75 *** | 0.56 | 0.94 |
| **100 *** | 0.56 | 0.94 |

* Due to efficiency of recycling recycled contents above 50% is not included in the LCA scenarios.

## Data providers

*Collection and sorting*

Collection and sorting data was provided by companies Pellenc, FiliGrade, and PAPREC. FiliGrade has developed a closed-loop sorting machine, including a Curve Code Reading System (CRS) [56]. NIR and DW technologies were used combined on a prototype made in collaboration with pellenc ST and Filigrade.

Different batches have been prepared according to the initial structure and origin of the multilayers (household or agriculture). For the agricultural films, BARBIER sampled them from its recycling plant and sent them directly to AIMPLAS for grinding without any sorting step. For the household films waste, PAPREC sampled it from three of its French MFRs and sent it to PELLENC ST. Film packaging from household waste collection has been sampled in three French MFRs: Rennes (Paprec Bretagne), Lyon (Paprec Trivalo 69), and Montpellier (Delta Recyclage). These MFRs have been chosen because they are run by PAPREC; they are designed to sort films and represent different locations (geographical and way of living). A fourth MRF in the North was supposed to be included, but its air separation process was out of order during the sampling period

As PAPREC has no agricultural films recycling plants for sampling, BARBIER and ADIVALOR helped for the definition of sample selection and how to sample it. After further discussion, the following was established:

- There are only simple multilayers (mechanically recyclable) in agricultural films EVOH/PE and PA/PE.
- They are used in only two kinds of applications: fumigation and silage films (collected separately).
- The proportion of these multilayer films in the French stream is very low because they are still in development, and the French design for recycling guidelines do not favour the use of these multilayers.
- The conclusion was that sampling silage and fumigation films from postconsumer collection would not guarantee the presence of multilayer films, or only very few.

*Pretreatment*

In order to maximize the surface area and increase the efficiency of the washing process, the samples were shredded to smaller sizes. For this purpose, a cutter equipment was used, obtaining strips of 15-20 mm in width.

The washing process was performed on household post-consumer waste, in a preindustrial washing tank at room temperature, using mild surfactant. The samples were rinsed inline, followed by a centrifuge to eliminate moisture. Before the rinsing step, water was left still to allow high-density fractions to sink (flotation step). No heavy fractions were detected for these materials. However, during the feeding of the material to the cutter and the washer, some contaminants were detected and manually separated. These were composed of expanded PE, aluminum foils and lids, paper linings, photographs, and sanitary face masks, among other products.

Pretreatment is carried out to eliminate residues of food and other contaminants. The pretreatment data used is based on the data reported by Franklin Associates and is similar for alternative and SoA scenarios [41].

## Dissolution

The data for dissolution was provided by TNO. The dissolution process in this study was developed by TNO, based on a similar concept from literature. Dissolution employs a solvent which dissolves exclusively polymers. Therefore, polyolefins (PO) can be separated as a pure stream. The Newcycling process, operated by APK AG, and the CreaSolv Process, operated by Unilever and the Fraunhofer Institute, use this technology for recycling of PO [18,20,21, 57-59].

The processing steps are as follows:

1. Dissolution (and coarse filtration).
2. Fine filtration.
3. Polymer precipitation and recovery.

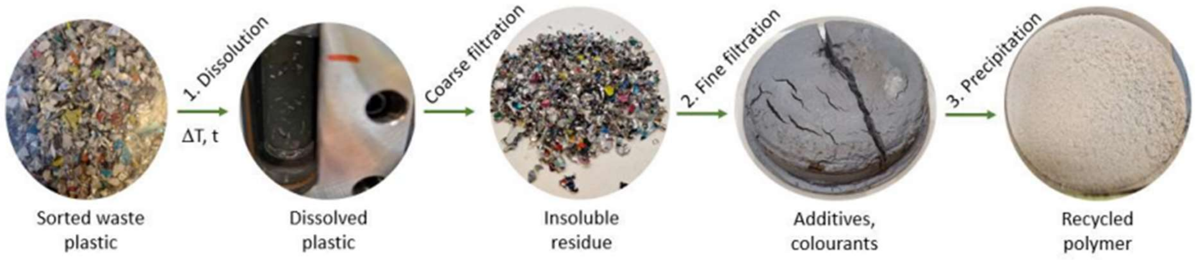


**Figure S2.** CIMPA processing scheme on sorted waste sample at lab scale.

The recycled PO material from real waste sample was analyzed using complementary analytical techniques available at TNO and VTT (such as FTIR, XRF, TGA, DSC) in order to determine the quality of the product and the amount of solvent remaining after the dissolution process of TNO. Thermal analysis of the product confirmed the removal of stabilizers that will need to be added before the recompounding step. No residual solvent was detected in the recycled product, confirming that a devolatilization step is not necessary after the physical recycling process, and the recovered material can be directly upgraded. Furthermore, the recyclate showed high PO purity with a low amount of residual impurities (such as, e.g., Ti, which comes from $TiO_2$ added to the foils typically in high quantities to make them white or intensely coloured).

In order to scale up the TNO's TRL3-4 technology for dissolution and realize a TRL5 laboratory pilot scale facility with the capacity to produce kg scale batches of recovered PO after dissolution, a PFD of the TR5 pilot was drawn (Figure S3).

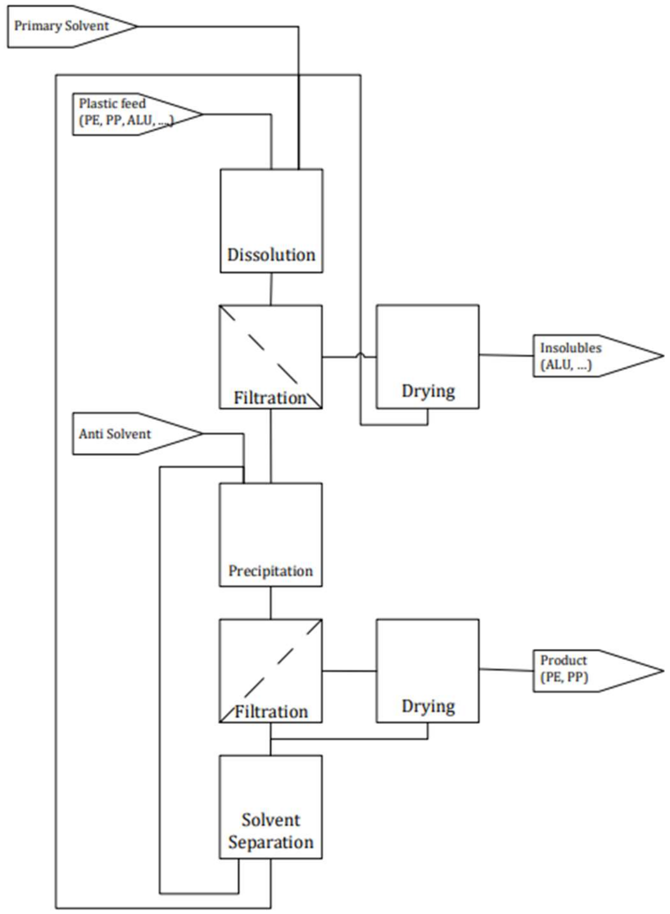


**Figure S3.** Process flow diagram of the TNO Möbius dissolution technology as designed for CIMPA.

## $scCO_2$ decontamination

The decontamination of the recycled plastic makes it possible to eliminate, or at least reduce, the presence of substances that may imply certain limitations [60]. AIMPLAS has patented a decontamination process consisting of a tandem single-screw extruders and supercritical $CO_2$ ($scCO_2$) as the stripping agent. This process has proved to be efficient in decontaminating plastic waste from organic contaminants and providing a quality which complies with the REACH regulations. The data for decontamination for this study was provided by AIMPLAS.

Detailed description of this process can be found at https://cimpa-h2020.eu/odour-removal-and-decontamination-of-multilayer-films/

*Extrusion with in-line rheology control*

Value Retention Extruder (VAREX) extrusion line is an advanced mechanical recycling technology developed by VTT [61,62]. This innovative tandem extrusion line upgrades the properties of mechanically recycled plastics using in-line measurements of melt rheological properties. VTT provided the extrusion data for the study.

Detailed description of this process can be found at https://cimpa-h2020.eu/in-line-rheology-control-and-upgrading-of-recycled-plastics/

*METEOR*

METEOR® is a unique process, developed and patented by IPC (FR3054159A1), which utilizes elongational mixing instead of the conventional shear mixing found in standard extruders. METEOR® is designed to enhance the dispersive mixing of polymer

blends, particularly those derived from the recycling sector. With its predominance of elongational flow mixing, surpasses the deformation and dispersion limits of shear flows. This capability enables the creation of added value by achieving fine nodular dispersions and fine elongated morphologies. Fine nodular morphology generally contributes to homogeneous physical bulk properties, while fine elongated morphologies enhance gas barrier properties, which are highly valued in packaging applications.

*MNL*

MNL is an industry-available process designed to increase the number of layers in a structure (up to more than 4000 layers), while maintaining the thickness of the film unchanged. The resulting layers are nanometric in size, offering advantageous properties, particularly in terms of oxygen or water barrier performance.

## Characterization

*Migration*

Global Migration test was performed according to the UNE-EN 1186 "Materials and articles in contact with foodstuffs—Plastics—Part 3: Test methods for overall migration in evaporable simulants", in EtOH95% at 40ºC for 10 days and in Iso-octane at 20ºC for 2 days.

A TVOC (Total Volatile Organic Compounds) test was performed according to VDA 277 "Laboratory Emission Testing of Organic Compounds" in a Head Space Chromatograph.

*Gloss:*

The typical standards for gloss measurements were as follows: ASTM D2457–08e1 Standard Test Method for Specular Gloss of Plastic Films and Solid Plastic.

*OTR:*

Oxygen transmission rate was measured using an OXTRAN ® 2-22L produced by the company MOCCON according to standard ASTM D3985. Two samples are analyzed at the same time and with a relative humidity of 0% at 23°C[63].

*WVTR:*

Water vapour transmission rate was measured using an PERMATRAN ® W-3-34H produced by the company MOCCON according to standard ISO-15106-2. Two samples are analyzed at the same time and with a relative humidity of 90% at 38°C.

*Tensile:*

Elongation at break and stress at break were measured using a 1ST traction bench from Tinius Olsen and using the ISO - 527 standard.

*Tear:*

Tear Strength was measured using a 1ST traction bench from Tinius Olsen and using the ISO – 6383 - 1 standard.

*Dart:*

Dart impact measurements were performed using the ISO 7765-1 standard.

## Material Circularity Indicator (MCI)

Material circularity indicator (MCI) calculations were performed based on the literature. Based on the reference, a sorting efficiency (after use and waste collection) is considered in calculations and defined as ES, while related waste is defined as WS.

Vadoudi et al. calculated the MCI for three loops of reprocessing PET/PU (tie layer)/PE film accounting for the quality of recycled plastic as X (utility factor) [37]. The amount of X in the first round of recycling is calculated as 1 (the industry average), where the fraction from virgin feedstock (V) is 100 %. By increasing the amount of fraction from recycled sources, the value of X rises above 1 and, consequently, the value of the MCI is closer to 1 (for the detailed calculations see Supporting Information.xlsx, tab MCI).

**Table S4**. MCI results for PET/LDPE sample. The results show the first life cycle.

| Adapted MCI | SoA | | CIMPA | | Reference [37] | |
|---|---|---|---|---|---|---|
| **Parameter** | **PET** | **LDPE** | **PET** | **LDPE** | **PET** | **LDPE** |
| **V (kg)** | 0.0168 | 0.065 | 0.017 | 0.065 | 0.247 | 0.730 |
| **$F_R$ (kg)** | 0 | 0 | 0.000 | 0.000 | 0 | 0 |
| **M (kg)** | 0.0168 | 0.065 | 0.017 | 0.065 | 0.247 | 0.730 |
| **$E_S$** | 0% | 0% | 55.2% | 55.2% | 58% | 58% |
| **$C_R$ (kg)** | 0 | 0 | 0.009 | 0.036 | 0.143 | 0.423 |
| **$W_S$ (kg)** | 0.0168 | 0.065 | 0.008 | 0.029 | 0.104 | 0.307 |
| **$E_C$** | 0 | 0 | 76.6% | 76.6% | 72% | 64% |
| **$W_C$ (kg)** | 0 | 0 | 0.002 | 0.008 | 0.040 | 0.152 |
| **W (kg)** | 0.0168 | 0.065 | 0.010 | 0.038 | 0.143 | 0.459 |
| **LFI** | 1 | 1 | 0.789 | 0.789 | 0.79 | 0.81 |
| **X** | 1 | 1 | 1.000 | 1.000 | 1 | 1 |
| **F(X)** | 0.9 | 0.9 | 0.900 | 0.900 | 0.9 | 0.9 |
| **MCI** | **0.1** | **0.1** | **0.290** | **0.290** | **0.29** | **0.27** |

**Table S5**. MCI results for PET/LDPE sample. The results show the second life cycle. The quality value and recycled input of 90% and 10% are used for CIMPA, respectively. The reference result is based on literature for 42% recycled content as the input to the second life cycle.

| Adapted MCI | CIMPA | | Reference | |
|---|---|---|---|---|
| **Parameter** | **PET** | **LDPE** | **PET** | **LDPE** |
| **V (kg)** | 0.015 | 0.059 | 0.143 | 0.459 |
| **$F_R$ (kg)** | 0.002 | 0.007 | 0.103 | 0.270 |
| **M (kg)** | 0.017 | 0.065 | 0.247 | 0.730 |
| **$E_S$** | 0.552 | 0.552 | 58% | 58% |
| **$C_R$ (kg)** | 0.009 | 0.036 | 0.060 | 0.157 |
| **$W_S$ (kg)** | 0.008 | 0.029 | 0.104 | 0.307 |
| **$E_C$** | 0.766 | 0.766 | 72% | 64% |
| **$W_C$ (kg)** | 0.002 | 0.008 | 0.017 | 0.057 |
| **W (kg)** | 0.002 | 0.008 | 0.144 | 0.459 |
| **LFI** | 0.010 | 0.038 | 0.58 | 0.63 |
| **X** | 0.739 | 0.739 | 1.42 | 1.37 |
| **F(X)** | 1.000 | 1.000 | 0.63 | 0.66 |
| **MCI** | 0.900 | 0.900 | 0.63 | 0.59 |
| **MCI** | **0.33** | | **0.59** | |

*Sources of electricity for 2050*

**Table S8.** Sources of electricity grid mix for the future scenario (2050) (International Energy Agency (2023)).

| Source | Percentage |
|---|---|
| **Total** | 100.00% |
| **Natural gas** | 17.04% |
| **Coal** | 11.77% |
| **Oil** | 1.45% |
| **Nuclear** | 22.58% |
| **Hydro** | 13.59% |
| **Geothermal** | 0.23% |
| **Wind Onshore** | 16.56% |
| **Wind Offshore** | 2.06% |
| **Solar** | 9.10% |
| **Biomass** | 5.25% |
| **Other renewable energies** | 0.00% |
| **Other fuels** | 0.37% |

*Demonstrator picture*

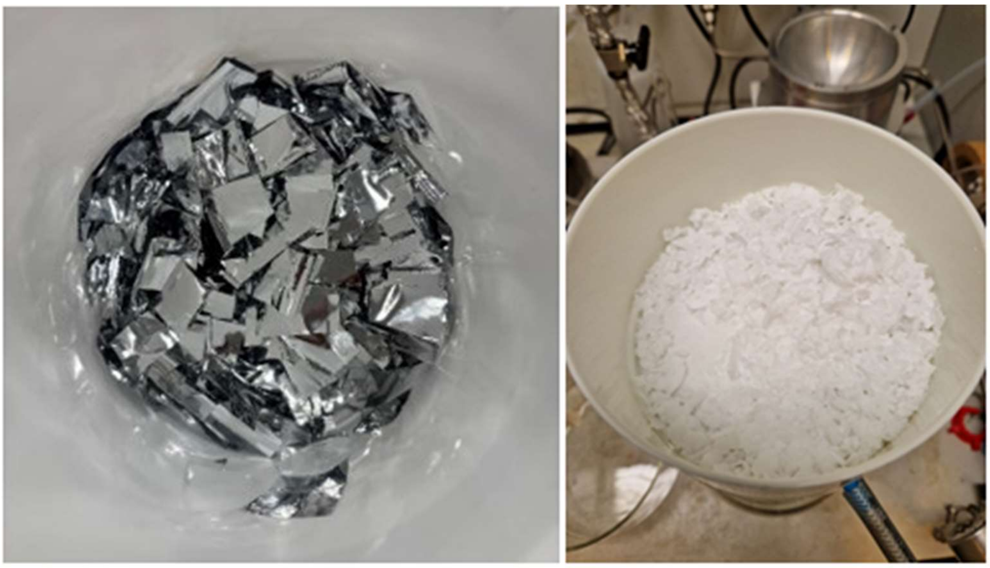

**Figure S4.** Demonstrators from dissolution experiments.

**Table S9.** Result of characterization for the four demonstrators.

| Properties | PET/PE | PE/PA | PE/EVOH | Metalized |
|---|---|---|---|---|
| **Tear Resistance MD (N/mm)** | 11.6 | 3.28 | 26.3 | 75.6 |
| **Resistance at break MD: UNE 53942 (MPa)** | 15.8 | 20.9 | 18 | 20 |
| **Elongation at break MD UNE 53942** | 510 | 390 | 480 | 698 |
| **Dart impact: ISO 7765-1 (g /µ)** | 0.53 | 0.54 | 2.54 | 2.97 |
| **Haze (%)** | 89.5 | 59 | 84.2 | 85 |
| **CoF** | 0.15 | 0.155 | 0.11 | |
| **Gloss (%)** | 62.4 | 46.15 | 52.9 | |
| **Odour** | No odour | No odour | No odour | No odour |

| | | | | |
|---|---|---|---|---|
| **WVTR (g/m2/day)** | 5.57 | 5.77 | 4.68 | |
| **OTR ((ml.cm)/(m2.day))** | 1566 | 1479 | 1515 | 3000 |
| **Migration test (NIAS)** | Passed | Passed | Passed | Passed |

**Table S10.** The eighteen categories that ReCiPe 2016 used at midpoint level [64].

| Entry | Midpoint Impact Category | Unit |
|---|---|---|
| **1** | Global warming | kg $CO_2$ eq |
| **2** | Stratospheric ozone depletion | kg CFC11 eq |
| **3** | Ionizing radiation | kBq Co-60 eq |
| **4** | Ozone formation, human health | kg NOx eq |
| **5** | Fine particulate matter formation | kg $PM_{2.5}$ eq |
| **6** | Ozone formation, terrestrial ecosystems | kg NOx eq |
| **7** | Terrestrial acidification | kg $SO_2$ eq |
| **8** | Freshwater eutrophication | kg P eq |
| **9** | Marine eutrophication | kg N eq |
| **10** | Terrestrial ecotoxicity | kg 1,4-DCB |
| **11** | Freshwater ecotoxicity | kg 1,4-DCB |
| **12** | Marine ecotoxicity | kg 1,4-DCB |
| **13** | Human carcinogenic toxicity | kg 1,4-DCB |
| **14** | Human non-carcinogenic toxicity | kg 1,4-DCB |
| **15** | Land use | $m^2$a crop eq |
| **16** | Mineral resource scarcity | kg Cu eq |
| **17** | Fossil resource scarcity | kg oil eq |
| **18** | Water consumption | $m^3$ |

## References


11. Antonopoulos, I.; Faraca, G.; Tonini, D. Recycling of post-consumer plastic packaging waste in the EU: Recovery rates, material flows, and barriers. *Waste Manag.* **2021**, *126*, 694–705. https://doi.org/https://doi.org/10.1016/j.wasman.2021.04.002.

18. Schwarz, A.E.; Ligthart, T.N.; Godoi Bizarro, D.; De Wild, P.; Vreugdenhil, B.; van Harmelen, T. Plastic recycling in a circular economy; determining environmental performance through an LCA matrix model approach. *Waste Manag.* **2021**, *121*, 331–342. https://doi.org/https://doi.org/10.1016/j.wasman.2020.12.020.

20. Walker, T.W.; Frelka, N.; Shen, Z.; Chew, A.K.; Banick, J.; Grey, S.; Kim, M.S.; Dumesic, J.A.; Van Lehn, R.C.; Huber, G.W. Recycling of multilayer plastic packaging materials by solvent-targeted recovery and precipitation. *Sci. Adv.* **2020**, *6*, eaba7599. https://doi.org/10.1126/sciadv.aba7599.

21. Berkane, I.; Cabanes, A.; Horodytska, O.; Aracil, I.; Fullana, A. The delamination of metalized multilayer flexible packaging using a microperforation technique. *Resour. Conserv. Recycl.* **2023**, *189*, 106744. https://doi.org/https://doi.org/10.1016/j.resconrec.2022.106744.

37. Vadoudi, K.; Deckers, P.; Demuytere, C.; Askanian, H.; Verney, V. Comparing a material circularity indicator to life cycle assessment: The case of a three-layer plastic packaging. *Sustain. Prod. Consum.* **2022**, *33*, 820–830. https://doi.org/https://doi.org/10.1016/j.spc.2022.08.004.

41. Franklin Associates. Life Cycle Impacts For Postconsumer Recycled Resins: PET, HDPE, and PP. 2018, *Submitted*.

56. FiliGrade. CurvCode. **2022**. https://www.curvcode.com/ (accessed on 6 August 2025).

57. ZZhao, Y.; Lv, X.; Ni, H. Solvent-based separation and recycling of waste plastics: A review. *Chemosphere*. **2018**, 209, 707–720. https://doi.org/10.1016/j.chemosphere.2018.06.095.

58. Georgiopoulou, I.; Pappa, G. D.; Vouyiouka, S. N.; Magoulas, K. Recycling of post-consumer multilayer Tetra Pak® packaging with the Selective Dissolution-Precipitation process. *Resour. Conserv. Recycl*. **2021**, 165, 105268. https://doi.org/10.1016/j.resconrec.2020.105268.

59. Aragonés, V. G. Odour removal and decontamination of multilayer films. **2022**. https://cimpa-h2020.eu/odour-removal-and-decontamination-of-multilayer-films/ (accessed on 6 August 2025).

60. Alassali, A.; Aboud, N.; Kuchta, K.; Jaeger, P.; Zeinolebadi, A. Assessment of Supercritical $CO_2$ Extraction as a Method for Plastic Waste Decontamination. *Polymers*. **2022**, 12, 1347. https://doi.org/10.3390/polym12061347.

61. Rytöluoto, I.; Pelto, J. VTT Polymer Pilot: Advanced Mechanical Recycling. **2022**. Available online: https://www.vttresearch.com/sites/default/files/2025-03/Advanced%20compounding%20and%20mechanical%20recycling%20pilot%20line.pdf (accessed on 6 August 2025).
62. Tenhunen, A.; Pöhler, H. A Circular Economy of Plastics: A vision of redesigning plastics value chains. **2020**, 24–25. https://doi.org/10.32040/2020.978-951-38-8824-4.
63. Messin, T.; Marais, S.; Follain, N.; Guinault, A.; Gaucher, V.; Delpouve, N.; Sollogoub C. Biodegradable PLA/PBS multinanolayer membrane with enhanced barrier performances. *J. Memb. Sci*. **2020**, 598, 117777. https://doi.org/10.1016/j.memsci.2019.117777.
64. Huijbregts, M.A.J.; Steinmann, Z.J.N.; Elshout, P.M.F.; Stam, G.; Verones, F.; Vieira, M.; Zijp, M.; Hollander, A.; van Zelm, R. ReCiPe2016: A Harmonised Life Cycle Impact Assessment Method at Midpoint and Endpoint Level. *Int. J. Life Cycle Assess*. **2017**, 22, 138–147, https://doi:10.1007/s11367-016-1246-y.

**Disclaimer/Publisher's Note:** The statements, opinions and data contained in all publications are solely those of the individual author(s) and contributor(s) and not of MDPI and/or the editor(s). MDPI and/or the editor(s) disclaim responsibility for any injury to people or property resulting from any ideas, methods, instructions or products referred to in the content.
